# Examining sedentary behaviours of adults with intellectual disabilities: A qualitative analysis

**DOI:** 10.1177/17446295241245784

**Published:** 2024-04-04

**Authors:** Sana Safi, Gina Wong, Lorraine Thirsk, Jeff Vallance

**Affiliations:** Faculty of Health Disciplines, 1349 Athabasca University, Athabasca, AB, Canada; Faculty of Health Disciplines, 1349 Athabasca University, Athabasca, AB, Canada; Faculty of Health Disciplines, 1349 Athabasca University, Athabasca, AB, Canada; Faculty of Health Disciplines, 1349 Athabasca University, Athabasca, AB, Canada

**Keywords:** adults, Critical Incident Technique, intellectual disability, qualitative research, sedentary behaviours

## Abstract

Sedentary behaviours among adults with intellectual disabilities have not been well studied. A sedentary lifestyle puts adults with and without disabilities at high risk of developing health conditions and diseases. Current literature revealed few empirical studies on the benefits of reducing sedentary behaviours with respect to the health of adults with intellectual disabilities. This research explored the factors that helped or hindered sedentary behaviours of adults with intellectual disabilities in the Canadian population. Guided by the socio-ecological model, Critical Incident Technique (CIT) was conducted. Five adults with intellectual disabilities from the Province of Ontario were interviewed and 102 critical incidents were collected. Adults with intellectual disabilities identified personal and environmental related factors that led to increased sedentary behaviours; and revealed helpful factors and wish-lists of actions that decreased sedentary lifestyle. Findings may be useful when developing programs aimed to decrease prolonged periods of sedentary behaviours specific to this vulnerable population.

## Introduction

Approximately 6.2 million Canadians who are 15 years of age and older live with a disability. Of these individuals, 1% live with intellectual disabilities (Statistics Canada, 2018). Intellectual disability can be defined as significant restrictions in an individual’s intellectual function and adaptive behaviours that begin before the age of 18 years (Chow et al., 2018; Harris et al., 2018). Intellectual disabilities include Fragile X syndrome, Down syndrome, developmental delay, Prader-Willi Syndrome, and Fetal Alcohol Spectrum Disorder (Mefford et al., 2012). Adults with disabilities tend to have poorer health behaviours which lead to diseases and health conditions (Agiovlasitis et al., 2020). Adults with intellectual disabilities spend an estimated 85% of their waking time in sedentary pursuits (Melville et al., 2015) compared to 65-80% sedentary time reported in adults without intellectual disabilities (Chastin et al., 2015). Sedentary behaviour (SB) can be described as a cluster of behaviours that appear in different contexts such as at home, school, or work, during leisure time, and while using transportation (Agiovlasitis et al., 2020; Harvey et al., 2013).

It is important to note that sedentary behaviour is different from physical inactivity. Physical inactivity can be defined as performing insufficient amounts of moderate-intensity to vigorous-intensity physical activity (MVPA), such as not meeting specified physical activity guidelines (Tremblay et al., 2017). The recommended daily activity for adults (ages 18 – 65) is 30-60 minutes of moderate activity five days per week, or 20-60 minutes of vigorous activity three days per week (Walsh et al., 2018). These recommendations include all adults with or without intellectual disabilities from the same age group. According to Walsh et al. (2018), if adults with intellectual disabilities cannot meet the daily activity guidelines due to their conditions, they should aim to be active in accordance with their ability to reduce the risk of developing health conditions or diseases.

Literature reviews suggested that only 9% of adults with intellectual disabilities meet the physical activity guidelines compared with 77% of the general population (Chow et al., 2018; Westrop et al., 2019). Melville et al. (2018) disclosed that 50% of the 725 adults with intellectual disabilities who participated in their study reported spending at least 4 hours a day in front of a screen. A comparative study by Dixon-Ibarra et al. (2013) indicated that adults with intellectual disabilities spent 61% of their waking time engaging in sedentary activity compared with 55% for adults without intellectual disabilities. A systematic review of 19 studies on SB of adults with intellectual disabilities revealed that sedentary time ranged from 522-643 min/day (Melville et al., 2017). Another systematic review on the prevalence of sedentary behaviours in older adults indicated that about 60% of this population is seated for more than 4 hours per day, where 54% of participants reported watching television for more than 3 hours and 65% spent over 3 hours in front of a screen (Harvey et al., 2013). Similarly, a recent systematic review of 17 studies that measured SB in individuals with Down syndrome indicated that sedentary time ranged between 392-680 min/day (Agiovlasitis et al., 2020). The results of these studies showed that adults with intellectual disabilities spend more than 1/3 of their waking time sedentary.

Among adults with intellectual disabilities, several factors were associated with spending extended periods in SB, such as social isolation (Melville et al., 2018). Mental health comorbidities such as anxiety and depression have been proposed to be linked to increased SB (Koyanagi et al., 2018; Melville et al., 2018). A comparative study by Dixon-Ibarra et al. (2013) on SB in adults with intellectual disabilities indicated that individuals of this population face many challenges that led them to be more sedentary than active. In addition, a study which measured sedentary behaviours in adults with intellectual disabilities reported that prolonged viewing of television and computers led to psychological issues due to an increase in isolation and a lack of social interaction with others (Harris et al., 2018). Furthermore, SB increase the likelihood of having multimorbidity conditions in adults with intellectual disabilities regardless of age, gender, walking ability, severity of intellectual disability, ethnicity, and socio-economic status (Tyrer et al., 2019). Dependence on family members or caregivers for help with activities of daily living, for example, led to increased SB in this population (Dixon-Ibarra et al., 2018).

Quantitative studies from different countries identified many barriers that lead adults with intellectual disabilities to adopt SB and refrain from participating in physical activities. Social isolation, experienced by many adults with intellectual disabilities is one barrier that results in extended periods of SB (Melville et al., 2018). Lack of interest, low self-efficacy, availability of resources, restrictive environments, and destructive support from caregivers are some other barriers that led to increased SB in this population (Dixon-Ibarra et al., 2018). Moreover, stigmatization and discrimination are key obstacles that may lead adults with intellectual disabilities to experience negative thoughts about themselves (Melville et al., 2018; Walsh et al., 2018). When faced with these barriers, the self-esteem of adults with intellectual disabilities may be affected, resulting in increased anxiety levels and a lack of participation in the community.

Economics and transportation further restrain adults with intellectual disabilities from becoming active, making them more likely to adopt SB (Dixon-Ibarra et al., 2018; Melville et al., 2015). Due to their cognitive limitations, adults with intellectual disabilities are not able to access public transportation without help from caregivers (Dixon-Ibarra et al., 2018; Melville et al., 2015). The Council of Canadians with Disabilities (n.d.) reported that individuals with disability live in poverty as they receive unequal education and have less opportunities for employment which furthers SB. A comparative study regarding sedentary behaviours between adults with and without intellectual disabilities found that adults with intellectual disabilities spent most of their waking time being sedentary regardless of age and gender (Oviedo et al., 2019). With the presence of these barriers, a very small number of adults with intellectual disabilities are able to be active in the community, whereas the vast majority are leading sedentary lifestyles.

Evidence from previous studies suggested that breaking up and reducing periods of SB decreases health risks in adults with intellectual disabilities (Oviedo et al., 2017). For instance, walking is the most affordable physical activity program that suits individuals with intellectual disabilities across their lifespan, which helps in the reduction of sedentary behaviours (Agiovlasitis et al., 2020; Chow et al., 2018). A comparative study on the level of physical activity and sedentary behaviours indicated that a small increase in daily physical activity provided significant increase in health outcomes for adults with intellectual disabilities (Dixon-Ibarra et al., 2013). A longitudinal investigation about the prevalence of SB in 1618 adults with intellectual disabilities by Hsieh et al. (2017) suggested that interventions to reduce sedentary behaviours in adults with intellectual disabilities should focus on disrupting periods of SB. The researchers in the above-mentioned study suggested that introducing light-intensity activities such as walking would help adults with intellectual disabilities to be less sedentary (Hsieh et al., 2017). Community activities such as organized outings, bowling, and park activities were some of the interventions that helped reduce sedentary behaviours in adults with intellectual disabilities (Hsieh et al., 2017).

Limited studies have been conducted examining this issue from the perspective of those living with intellectual disabilities. Thus, conducting a qualitative study using the Critical Incident Technique guided by the socio-ecological model helped investigate and analyze SB of adults with intellectual disabilities by determining what aids or hinders activity levels in the population. Further in-depth investigation into the factors that led to increased or decreased SB of adults with intellectual disabilities in the Canadian population is needed to better support and improve their health outcomes. The purpose of this research was to examine SB patterns of adults with intellectual disabilities in Ontario, Canada.

## Method

### Design overview and population

Critical Incident Technique (CIT) was used to explore SB patterns among adults with intellectual disabilities. Critical Incident Technique can be defined as the process of collecting critical factors and facts regarding a specific behaviour in a defined condition (Viergever, 2019). Critical Incident Technique is considered the newest methodology in qualitative research and can be used to examine under-studied issues or incidents that are affected by the different factors (Butterfield et al., 2009). Critical Incident Technique can be described as an inductive approach that focuses on the participants’ perspectives and opinions regarding a specific experience or incident (Persolja, 2020; Viergever, 2019). The “critical incident” was used as a unit of analysis when analyzing and interpreting data in CIT (Butterfield et al., 2009). Five adults with intellectual disabilities from the Province of Ontario were interviewed. Adults aged 18 - 65 years old who were diagnosed with intellectual disabilities were invited to participate in the research. The research included participants of various genders, ethnicities, and levels of education.

### Inclusion and exclusion criteria

The inclusion criteria included adults diagnosed with mild intellectual disabilities who identified themselves as living a sedentary lifestyle. Participants of any gender who met the age range of 18-65 and who were living in Ontario, Canada were sought. Participant involvement was up to two hours distributed across two virtual interviews. Participants included in the study were able to speak and comprehend the English language. The exclusion criteria included adults unable to communicate verbally. Adults using augmented and alternative communication devices (AAC), individuals younger than 18 years of age or older than 65, and non-English speakers, were also excluded.

### Sampling

Purposive sampling was used to involve participants who met inclusion criteria for this study. According to Butterfield et al. (2009) there is no specific sample size required in CIT in comparison to other qualitative research methodologies. The researcher should continue interviewing participants until they reach data saturation. Data saturation can be achieved by collecting between 50-100 critical incidents for simple activities or several thousand critical incidents for more complex activities (Butterfield et al., 2009). Data saturation can also be reached when no new categories occur to describe the critical incidents (Viergever, 2019). In CIT, recruiting small sample size can be sufficient considering the methodology’s rapid data saturation (Butterfield et al., 2009). Initially, 7 participants were interviewed; however, two participants were excluded (N=5) for not meeting the inclusive criteria. That is, one was excluded as they did not reside in Ontario, and the other was excluded because they were non-verbal. A total of 102 critical incidents were collected from interviewing the five participants.

### Data collection

Data collection occurred through in-depth semi-structured interviews. Virtual interviews were conducted in adherence to the COVID-19 restrictions placed by the Ethics Board at the time of conducting the research, as well as in consideration for the participants’ financial and social difficulties. The Zoom Video Communication website was used to conduct and record the interviews with all participants. According to Gray et al. (2020), the advantages of using online interviews include saving travel costs, reducing time in data transcription, creating a safe and comfortable environment for participants, and enabling participants to discuss more sensitive issues. The data was collected during the COVID-19 pandemic between the months of July and October of 2021.

Interview questions focused on identifying factors, supports, and beliefs which led to living a sedentary lifestyle for adults with intellectual disabilities. Participants were asked to describe their insights of being either active or inactive. The interview questions were structured into a list of key questions and prompt questions. The interview began with a key question followed by numerous related prompts. The prompt questions allowed participants to elaborate on earlier responses, enriching the quality of collected data. During the interviews, open-ended and non-directional questions were used to enable an in-depth dialogue to help participants share their views and insights regarding sedentary lifestyle (Cunningham et al., 2020). Critical Incident Technique research also includes asking participants to share any wish list factors of becoming less sedentary (Viergever, 2019).

### Data analysis

Overall, 102 critical incidents were extracted and organized into codes using thematic analysis. In addition, Microsoft Word was used to transcribe the interviews and the researcher used highlighters to track codes. Codes were transferred into Excel spreadsheet to analyze and organize the data produced from the interviews into categories or themes. Using inductive reasoning, the researcher distinguished patterns, themes, similarities, or differences in the incidents provided by the participants to form categories (Clark and Veale, 2018; Viergever, 2019).

## Results

### Participants

Participants included 1 male and 4 females with the age range of 28 – 45 years. All participants had a diagnosis of mild intellectual disabilities and completed post-secondary education. At the time of data collection, 2 participants were employed and 3 participants were unemployed. All participants lived in the province of Ontario, where 3 participants lived in an apartment and 2 lived in a house. Two of the participants lived alone and 3 lived with family members.

### Emerging themes

The following four overarching themes emerged from data extraction of 102 critical incidents: (1) personal related incidents led to increase SB, (2) environmental related incidents aided to increase in SB (3) helpful incidents led to decrease SB, (4) wish-lists including participants’ thoughts and opinions of ways to becoming less sedentary.

#### Personal related incidents

Personal related incidents were described as internal events that led participants to become more sedentary including: leisure time, hobbies, activities of daily living, and health conditions. Participants reported spending most of their leisure time in front of a screen. Participants stated they spend hours watching TV, gaming, texting, and searching online on a daily basis. A participant stated “Some days I get lost in the world of video games. I try to set timers. I do not check the time and it has been 6 hrs.” All participants reported spending between 310-615 min/day in front of a screen. Participants reported reading, arts and crafts, knitting, and listening to music while laying down as hobbies. A participant stated “I read 30-180 min/day; not just in one sitting. Like 6 hours a week.” On average, participants spent between 30-270 min/day engaging in these hobbies.

Participants reported engagement in housekeeping activities once or twice a week whereas laundry was done once a week. Participants indicated taking breaks while cleaning or doing laundry by watching TV or working on a computer. One participant reported difficulty engaging in housekeeping activities due to left-sided weakness and dependency on caregivers. The participant stated, “Usually my mom or dad fix me my coffee and oatmeal. I fix toast on Sunday morning, but sometimes I burn them.” Most of the participants nap between 45-180 min/day which is considered SB. Chronic pain, weakness, seizure, and obesity led some of the participants to adopt a sedentary lifestyle. Several of the participants stated they could not continue with their daily activity due to health conditions. For example, one participant shared “If I have had a seizure, then obviously, it is game over.” Due to obesity, some participants chose to stay home as they felt tired constantly.

#### Environmental related incidents

Environmental related incidents were described as external events that aided to increase SB of adults with intellectual disabilities including: financial hardship, discrimination, safety, weather, lack of social support, distance, and noise levels. The COVID-19 pandemic was another environmental related incident that led to increase SB of adults with intellectual disabilities at the time of the study. Financial hardship was the main environmental related incident that led participants to become more sedentary. Most adults with intellectual disabilities relied on the Ontario Disability Support Program (ODSP) for living. Participants considered themselves poor because they lived primarily on ODSP, and many reported having no opportunities for full-time job positions. One participant stated “There is a gym near me which I can easily walk to but it is really expensive. You need money to build muscles.”

Participants raised concerns regarding safety and the need to have someone accompany them when going for walks in the community. They preferred staying at home than going outside because “people stare” as one of the participants stated, referring to their disability. Weather was also indicated as a deterrent to participate in different outdoor activities particularly for those who use mobility devices. Cold temperatures, piles of snow, and icy sidewalks hindered participants from engaging in community walks. Likewise, distance from gyms or community centres was a key hindrance for some participants. One participant was able to exercise on a daily basis because they had a gym in their family house basement. That participant said, “I work out every day at home. I head downstairs for another workout.” One of the participants stopped going to the gym due to increased sensitivity to noises.

In regards to COVID-19, all participants in the study reported decreased levels of activity related directly to the pandemic. Participants reported increased feelings of isolation due to a decrease in social interaction, increased agitation, fear, and panic attacks in regard to the COVID-19 pandemic. One participant described the situation during the COVID-19 pandemic by saying “I am just not fully satisfied because I do not have any social contact. My friends are too afraid to come near me anymore.” At the time of data collection, 4 of the participants were double vaccinated and one has a medical condition that prevented them from getting the COVID-19 vaccine.

#### Helpful incidents

Helpful incidents and/or activities were events described to support participants to become less sedentary. These included: outdoor walking, camping, indoor activities, use of fitness tracking device, and presence of a caregiver. All participants reported walking on a weekly basis either in the community and parks or to a nearby mall or plaza. Another outdoor activity was camping. One participant emphasized “I am a camp person. We are outside all day on summer camp. I can live there. I totally would.” Participants listed rugby and wheelchair basketball as indoor sports that they enjoyed before the COVID-19 pandemic. Some participants stated they engaged in some indoor activities such as a balancing and strengthening exercise and restorative yoga. Two participants reported using fitness tracking devices such as Da Fit and Fitbit to become more active. A participant stated “Fitbit will vibrate so I will get up and like take two steps and then that counts for something. I got one specially for COVID.”

Some participants shared that they depended on a private Personal Support Worker (PSW) or a Respite Worker (RW) to go for a walk outside in nature, or to attend some activities and sports. One participant stated, “I like to go to respite, this was pre-COVID. I do have a Respite Worker and we go for drives to get ice cream or we go to the mall. Respite Worker that does like. let’s go to hockey games or something like that.”

#### Wish-lists/participant suggested incidents

Wish-lists or participant suggested incidents are items and actions desired by the participants to aid in becoming less sedentary. The list included: providing resources, education, accessibility, and increased financial support. Participants identified resources as social stories, sensory tools, posters, and prizes. Examples of simple prizes that were suggested by adults with intellectual disabilities themselves include colouring books and crayons, mini-individualized whiteboards, or a squishy ball. Participants proposed that raising awareness and education were two contributors in becoming less sedentary. Participants highlighted accessibility as a vital action to becoming less sedentary and suggested the need for transportation so that gyms could be more accessible.

Participants suggested increase financial support as one participant stated “I think ODSP need to go up. If ODSP does get increased and get the basic income, yes, I might try dancing again or I might become more active.” Some participants believed that money was the solution for many obstacles of becoming less sedentary. A participant stated “I think if I have more money, some things become more accessible in the moment.”

## Discussion

This research explored the factors, supports and beliefs that led to increased or decreased SB of adults with intellectual disabilities living in Ontario, Canada using Critical Incident Technique guided by the socio-ecological model. The demographics of participants such as their health condition, the lack of social support, and the environment led to the adaptation of SB in adults with intellectual disabilities. This research demonstrated that factors led adults with intellectual disabilities to become more sedentary could be related to participants themselves or internal incidents, or to the environment or external incidents. Also, the research identified support incidents that helped decrease SB of adults with intellectual disabilities. The research provided adults with intellectual disabilities the opportunity to provide wish-lists of actions to support them in becoming less sedentary.

Internal incidents leading to increased SB found in this research were consistent with other findings in the literature as adults with intellectual disabilities pass most of their leisure time in front of a screen, engaged in sedentary activities such as watching TV, gaming, texting, or searching online (Agiovlasitis et al., 2020; Melville et al., 2018). On average, adults with intellectual disabilities in this research spent 310–615 min in front of a screen on a daily basis. Reading was indented as the hobby of choice for adults with intellectual disabilities, which requires sitting or reclining for long periods of time. All other hobbies such as arts and craft, knitting, and listening to music while sitting were considered sedentary activities. In this research, adults with intellectual disabilities spent more than 4 hrs/d engaging in activities that required sitting which has also been reported in Harvey et al.’s systematic review (2013).

This research indicated that adults with intellectual disabilities tend to cook simple meals or purchase ready-to-eat meals, nap up to 3 hrs/day, and watch TV while waiting for their laundry which contributed to increase SB. Chronic pain, weakness, and obesity that were reported as hindrances could be related to being sedentary for most of the waking hours in adults with intellectual disabilities (Agiovlasitis et al., 2020; Melville et al., 2018). Other health conditions reported in this research, which contributed to increased likelihood of becoming more sedentary, include episodes of seizure, depression, and anxiety (Koyanagi et al., 2018; Westrop et al., 2019).

External incidents that led adults with intellectual disabilities to increase SB such as, financial hardship, discrimination, safety, weather, lack of social support, distance, noise levels and the COVID-19 pandemic identified in this research as well as others (Dixon-Ibarra et al., 2018; Melville et al., 2018). The outcomes of the interviews revealed that many adults with intellectual disabilities living in Ontario rely on ODSP which does not cover all basic needs. Adults with intellectual disabilities live in poverty as they rely on ODSP and they face difficulties finding jobs in general (Ministry of Children, Community and Social Services, 2022; Statistics Canada, 2022). According to Melville et al. (2018) there is a negative relationship between the severity of intellectual disabilities and SB which is apparent as all participants in this research have mild intellectual disabilities and reported spending up to 10 hrs/day engaging in SB.

The interviews indicated that some of the reasons for choosing to be at home was to avoiding discrimination, saving money, and lacking social support that appears critical in encouraging adults with intellectual disabilities to be more active inside and outside of their homes (Council of Canadians with Disabilities, n.d; Melville et al., 2015). Adults with mild intellectual disabilities understand discrimination, ableism, sexism, and Islamophobic attitudes directed towards them (Melville et al., 2018; Oviedo et al., 2019). Many may prefer to stay home to avoid experiencing these adversities, especially when they do not have access to an adult without a disability to ensure their safety. Sedentary behaviours appear to be a by-product of a defense mechanism adults with intellectual disabilities embody in order to feel safe (Agiovlasitis et al., 2020; Koyanagi et al., 2018).

The COVID-19 pandemic restrictions, the fear of getting COVID-19, or becoming exposed led adults with intellectual disabilities to stay home and engage in SB. The fear and nervousness of getting COVID-19 also contributed to increased anxiety, depression and isolation due to decrease in social interaction (Agiovlasitis et al., 2020; Gray et al., 2020). Also, adults with intellectual disabilities became more sedentary when the COVID-19 restrictions resulted in sudden loss of social support and a switching of in-person activities to fully virtual settings.

Some incidents helped adults with intellectual disabilities become less sedentary. These included walking, sports, and having social support (Melville et al., 2015; Tyrer et al., 2019). Outdoor activities such as walking in the community, to the convenient store, or as part of a summer camp helped adults with intellectual disabilities to decrease SB. This was the case for adults with intellectual disabilities who had a Personal Support Worker or a Respite Worker, not for those who lived by themselves. Walking is the most affordable exercise for adults with intellectual disabilities, but some hindrances play a part in making it difficult. Safety, weather, comorbidities, lack of social support and, in some situations, high levels of noise were some of the hindrances that made walking not a helpful incident for adults with intellectual disabilities (Dixon-Ibarra et al., 2018).

None of the adults with intellectual disabilities reported participating in kayaking, biking, or hiking as outdoor activities when asked during the interviews despite these activities being popular in Ontario. This could be due to poverty, lack of social support, distance, and health conditions. Most of these outdoor activities, if not all of them, cost money for training, equipment, and transport to a specific location. Some indoor sports such as rugby and wheelchair basketball that are offered to adults with intellectual disabilities helped them decrease their SB. These sports and other indoor activity programs such as community yoga classes, balancing and strengthening exercise programs, and home setting restorative yoga were incidents that helped adults with intellectual disabilities live a less sedentary lifestyle (Chow et al., 2018). Looking to the indoor activity programs that helped adults with intellectual disabilities to be less sedentary, it is clear that they were accessible largely due to the presence of financial and social support.

When asked ‘how can professionals and researchers help?’ adults with intellectual disabilities pointed out the need to increase and enhance knowledge and education regarding SB in a way that would be accessible to them. Adults with intellectual disabilities implied that the amount of literature available about SB is not sufficiently accessible to their specific population. The literature and information available do not provide a clear explanation about SB and its effect of the health and well-being of adults with intellectual disabilities. It appeared that adults with intellectual disabilities may be misunderstanding the meaning of SB, as they reported being ‘active’ simply because they spend hours during the day engaging in any activity such as using a screen, knitting, reading or listening to music while sitting for hours. They did not appear to understand that all of these activities are considered SB due to their low energy expenditure. Participants advocated to have free online websites or apps that can help them understand SB and how to decrease it through easy and affordable ways.

Increasing accessibility to facilities where adults with intellectual disabilities can be less sedentary was another suggestion. Accessibility should include the place, transportation, and fees or memberships (Melville et al., 2018; Melville et al., 2015). The place where adults with intellectual disabilities could be active should refrain from loud music or noise, minimize crowding, and use nature lights. In some cities in Ontario, public transportation should provide more services and stations so adults with intellectual disabilities would not have to walk for longer distances. Gym’s memberships and activity programs’ fees should be subsidized or discounted for adults with intellectual disabilities. Considering most adults with intellectual disabilities live in poverty as they receive ODSP and may be working in a casual position or be unemployed, they will highly benefit from these discounts. As per Maslow’s hierarchy of needs (Maslow, 1943), individuals focus on meeting physiological needs such as food, shelter, and clothing before focusing on safety needs such as health and personal security. Thus, there is a massive need for more investments in education and finances for adults with intellectual disabilities.

### Research strengths and limitations

The use of CIT enhanced the strength of the research as it provided an emphasis on incidents that led to increase or helped decrease SB among adults with intellectual disabilities. The focused sample size used in this research is another strength as it allowed for more in-depth understanding of the what-and-why questions regarding SB of adults with intellectual disabilities. Using semi-structured interviews permitted adults with intellectual disabilities to elaborate on their answers when sharing their thought, views, and beliefs regarding SB. Another strength in this research is the fact that adults with mild intellectual disabilities were offered the opportunity to share their thoughts and opinions regarding their living condition. All collected data originated from adults with mild intellectual disabilities themselves, unlike the more popular method of relying on a family member or a caregiver to communicate on behalf of the person with intellectual disabilities.

Some of the research limitations include participants’ improper understanding of SB that may have contributed to insufficient reporting, missing information, and lack of follow-up measurements. While the interviews were successfully conducted in a virtual environment due to the COIVD-19 pandemic restrictions, in-person interviews remain important as they capture non-verbal cues and non-spoken language. Hands gestures, head movement, facial expression, and body posture are non-verbal cues that help with data collection while conducting qualitative research. The time allocated for the research was not sufficient to conduct more follow-up sessions with participants. Small sample size due to the exclusion criteria and difficulties with the recruitment process. Recruitment was done electronically due to COVID-19 restrictions through the organization website and social media platforms. As a result, adults with intellectual disabilities who are not active on social media or do not have access to electronic devices did not receive the recruitment poster. Therefore, only a selective group of adults with intellectual disabilities were able to participate in the study. This led to a relatively small size sample with specific lifestyle, which may not represent all adults with mild intellectual disabilities. As this study was exploratory and included only adults with mild intellectual disabilities, the results cannot be generalized among the entire population of adults with intellectual disabilities. In light of the abovementioned limitations, more studies on the nature and reasons of the abundance of SB in adults with intellectual disabilities is needed.

### Implications for practice/policy

While people with intellectual disabilities face many of the same challenges that other people do in reducing sedentary behavior, this research demonstrated additional constraints such as income and poverty, experiences of stigma, and concerns about safety. With their focus on health promotion, understanding of the social determinants of health, and ethical obligation for advocacy, nurses in a variety of settings should be knowledgeable about intellectual disabilities and have the competencies to effectively work with this population. For all professionals who work in health promotion, strategies should not only include individually tailored interventions, but also advocacy for policy changes at the system level that would enable people with intellectual disabilities to reduce sedentary behavior more easily. Further, it is essential to advocate for the inclusion of adults with intellectual disabilities in conversations that will shape programs, policies, and services affecting this population.

## Conclusion

This research explored the views, perceptions and beliefs of adults with intellectual disabilities living in Ontario, Canada regarding their levels of SB. The research question focused on investigating the factors led to increased or decreased SB specific to this population. This research concluded that four main incidents played a big role in increasing or decreasing SB. Personal related incidents, environmental related incidents, helpful incidents, and recommendations identified by adults with intellectual disabilities were all incidents that enhanced or reduced SB. This research also concluded that adults with mild intellectual disabilities are capable of speaking for themselves, answering questions, and engaging in virtual interviews similar to adults without intellectual disabilities. They can be direct participants in research instead of relying on their support workers or family members to share their views and beliefs. They were able to provide valuable suggestions and recommendations that assisted and aided them to become less sedentary.
